# Risk Factors for Severe Hypocalcemia in Patients with Secondary Hyperparathyroidism after Total Parathyroidectomy

**DOI:** 10.1155/2021/6613659

**Published:** 2021-04-02

**Authors:** Ping Wen, Lingling Xu, Shasha Zhao, Wei Gan, Dawei Hou, Liang Zhang, Jinlong Cao, Mingxia Xiong, Lei Jiang, Junwei Yang

**Affiliations:** ^1^Center for Kidney Disease, The Second Affiliated Hospital of Nanjing Medical University, Nanjing, China; ^2^Department of General Surgery, The Second Affiliated Hospital of Nanjing Medical University, Nanjing, China

## Abstract

**Background:**

Hypocalcemia is the most common complication of total parathyroidectomy in secondary hyperparathyroidism (SHPT) and is associated with adverse consequences such as spasms, epilepsy, and arrhythmia and even death if the serum calcium level decreases rapidly. Previous studies have identified several risk factors for postoperative severe hypocalcemia (SH) in patients with SHPT, but the sample sizes were small and thus the results may not be reliable.

**Objectives:**

This study was performed to investigate the risk factors for SH after total parathyroidectomy without autotransplantation (tPTX) in a large sample of patients with uremic hyperparathyroidism.

**Methods:**

We retrospectively investigated the records of 1,095 patients with SHPT treated with tPTX between January 2008 and December 2018. Based on the postoperative serum calcium concentration, the patients were grouped into SH and non-SH groups. The clinical characteristics and biochemical results were analyzed, and binary logistic regression analysis was used to identify the risk factors for SH.

**Results:**

After surgery, 25.9% of the patients developed SH. Age, diastolic blood pressure (DBP), heart rate, frequency of bone pain, weight of resected glands, preoperative serum calcium, intact parathyroid hormone (iPTH), alkaline phosphatase (ALP), and hemoglobin levels differed between the two groups. Binary logistic regression analyses identified preoperative serum calcium, iPTH, and ALP levels as independent predictors of SH after surgery.

**Conclusions:**

The preoperative serum calcium, iPTH, and ALP levels can be used to assess the risk of postoperative SH in patients with SHPT. Such patients should thus be monitored closely in order to initiate prompt interventions to avoid SH.

## 1. Introduction

Secondary hyperparathyroidism (SHPT) is a major chronic complication in patients undergoing maintenance hemodialysis and is correlated with morbidity and mortality. SHPT is a chronic kidney disease- (CKD-) mineral and bone disorder and is characterized by alterations in serum calcium, phosphate, intact parathyroid hormone (iPTH), vitamin D, and FGF23; bone abnormalities; and vascular calcification. Medical treatment with vitamin D analogs can control this disease at an early stage. However, drugs may have little effect in the late stage, while nodular hyperplasia is the main pathological manifestation of the parathyroid glands. Although cinacalcet can provide a new strategy for patients with SHPT [1, 2], this agent can induce severe hypocalcemia (SH) or hypophosphatemia, which also requires a reduced dose or the use of other measures to control them. In addition, the expensive price limited the application of cinacalcet. Therefore, parathyroidectomy remains a valuable and useful method to treat severe SHPT, particularly in patients who are resistant to medical treatments or who cannot afford expensive agents.

Successful parathyroidectomy can have a striking effect in reducing the serum iPTH level but is associated with certain complications, among which SH is the most frequent. Postoperative SH is a well-known and severe complication following parathyroidectomy [3]. The reported incidence of postoperative SH is 10–46% among patients with primary hyperparathyroidism who underwent parathyroidectomy and 28–88% among patients with SHPT [4–9]. SH following parathyroidectomy can be severe and prolonged in some situations and a poorly defined entity termed “hungry bone syndrome” (HBS) has been adapted to describe this phenomenon. Old age, the size of the resected parathyroid glands, the preoperative serum iPTH level, and the preoperative serum alkaline phosphatase (ALP) level were found to be risk factors for SH after parathyroidectomy in patients with primary hyperparathyroidism [10–13]. Also, there is a study with controversial results that indicated young age was a risk factor for HBS [14]. Further, the preoperative iPTH and ALP levels were reported as risk factors for SH after parathyroidectomy in SHPT patients. However, the sample sizes of these studies were small. Hence, we conducted this study with a larger sample to provide more solid evidence regarding the clinical factors predictive of SH after total parathyroidectomy in patients with uremic hyperparathyroidism.

## 2. Materials and Methods

### 2.1. Study Population

Between January 2010 and December 2018, 1,095 patients with uremic hyperparathyroidism who underwent total parathyroidectomy were retrospectively reviewed in detail at the kidney center of the Second Affiliated Hospital of Nanjing Medical University. Clinical and biochemical data were collected from their electronic medical records. Indications for parathyroidectomy included at least one of the follows: (1) severe SHPT (persistent iPTH of >800 pg/mL) resistant to medical therapy with calcitriol or vitamin D analogs, (2) severe SHPT with hyperphosphatemia (>2.0 mmol/L), (3) severe SHPT with unbearable pruritus and/or bone pain, and (4) imagological examination showing hyperplasia of the parathyroid glands (>1.0 cm in diameter). The study was approved by the ethical committee of the Second Affiliated Hospital of Nanjing Medical University (ethical number: 2018KY110) and informed consent was obtained from all the patients.

### 2.2. Pre- and Postoperative Evaluations

Before surgery, routine blood tests were conducted, biochemical indices including liver function and electrolytes were assessed, iPTH and ALP levels were measured, and cardiac function and calcification were evaluated by echocardiography and X-ray. The calcium level was adjusted with serum albumin level. Aortic calcification includes thoracic and abdominal calcification was evaluated by X-ray before surgery. Bone pain was evaluated by visual analogue scale (VAS). For all patients, the dialysate calcium concentration was 1.5 mmol/L before surgery. Ultrasound and isotope tagging were applied to localize the parathyroid glands.

Postoperatively, the patients were administered a routine intravenous infusion of calcium gluconate via a central vein catheter. Oral calcium carbonate was supplemented from the second day after surgery. Serum iPTH levels were recorded at 1 h and 24 h after surgery. Serum calcium and phosphorous levels were monitored immediately after surgery and every day postoperatively. All glands removed from the patients were measured, weighed, and verified histologically. The patients were grouped into SH (calcium < 1.78 mmol/L) and non-SH (calcium ≥ 1.78 mmol/L) groups according to the postoperative serum calcium concentration.

### 2.3. Surgical Procedure

Because of the high recurrence rate, total parathyroidectomy without autotransplantation (tPTX) was performed in all patients included in this study. All patients underwent bilateral neck exploration with an attempt to identify all parathyroid glands. If four or more glands were identified, tPTX was performed. If fewer than four glands were found, all identifiable glands were removed.

### 2.4. Statistical Analysis

Statistical analyses of the collected data were performed using SPSS version 21.0. Data are expressed as the mean ± standard deviation if normally distributed, or the median and interquartile range otherwise. In the univariate analysis, categorical variables were compared using the chi-square test or Fisher's exact test, where appropriate. Continuous variables were compared using an independent sample *t*-test or the Wilcoxon rank-sum test for data of nonnormal distribution. Binary logistic regression analysis was used to identify the independent predictors of SH after surgery. Variables with a *p* value of <0.05 in the univariate analysis were entered into the binary logistic regression model. Variables with *p* values of <0.05 (two-tailed) were considered statistically significant.

A diagnostic model was tested including all available predictor variables (preoperative serum calcium and iPTH and ALP levels) using a forward-entry stepwise procedure with a threshold of *p* < 0.1 to enter the regression equation.

## 3. Results

The clinical and biochemical parameters of the study participants are summarized in Table 1. The mean age of the patients was 47.26 ± 10.86 years. All patients were on hemodialysis, and the median duration of dialysis was 84 months. The underlying renal diseases included chronic glomerular nephritis (58.9%), diabetic nephropathy (2.0%), hypertensive nephropathy (6.1%), polycystic kidney disease (5.2%), and others (6.9%, obstructive nephropathy, anaphylactic purpura nephritis, and systemic lupus erythematosus nephritis). However, 20.8% of the patients had unknown underlying renal diseases. Among all 1,095 patients, only 4.1% had diabetes and 79.6% had hypertension. The most common clinical symptoms were bone pain (79.7%) and pruritus (54.0%). Before surgery, the median iPTH level was 1,448.4 pg/mL (996.0∼2,173.8), which decreased to 2.8 pg/mL after tPTX. The preoperative serum phosphorus and calcium concentrations were 2.25 mmol/L and 2.43 mmol/L, respectively. The median ALP level was 205.6 U/L (117.0∼464.9) before the operation. The mean hemoglobin level was 107.67 g/L and 54.3% of the patients were found to have anemia. The mean serum calcium and phosphorus levels were 2.05 mmol/L and 1.80 mmol/L, respectively, on the first day after surgery, showing a significant reduction compared to preoperative levels. All patients underwent tPTX, and 90% of the resected glands had nodular hyperplasia.

**Figure 1:**
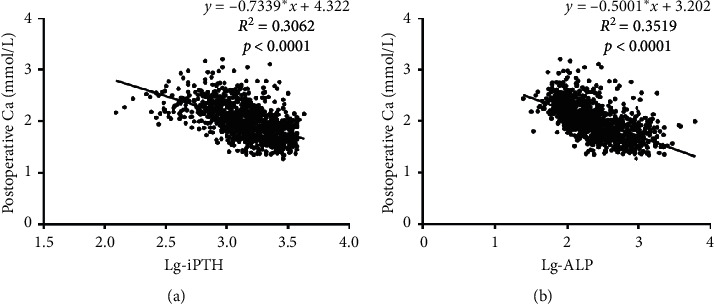
Linear regression of Lg-iPTH, Lg-ALP, and postoperative serum calcium. The linear regression analysis showed Lg-iPTH (a) and Lg-ALP (b) were negatively correlated with postoperative calcium and the correlation coefficients were 0.3062 (*p* < 0.0001) and 0.3519 (*p* < 0.0001).

As aforementioned, the patients were divided into two groups according to the postoperative serum albumin-adjusted calcium concentration: SH (calcium < 1.78 mmol/L) and non-SH (calcium ≥ 1.78 mmol/L). A total of 284 patients (25.9%) developed SH after tPTX. As shown in Table 2, the patients in the SH group were younger (44.6 ± 10.7 vs. 48.2 ± 10.8, *p* < 0.001), had higher diastolic blood pressure (DBP, 90.1 vs. 87.3 mmHg, *p*=0.007), had a faster heart rate (84.8 vs 82.2 bpm, *p*=0.001), and had more frequent bone pain (87.3 vs. 77.1%, *p* < 0.001) than the non-SH group. The preoperative laboratory tests revealed significant differences in iPTH, ALP, hemoglobin, and calcium levels between the two groups.  Figure 1 shows the linear correlations between Lg-iPTH, Lg-ALP (Lg: logarithm function), and postoperative serum calcium. Lg-iPTH and Lg-ALP were negatively correlated with postoperative calcium. The mean weight of the resected glands was significantly higher in the SH group than in the non-SH group (3.2 g vs. 2.4 g, *p* < 0.001). There were no differences in the rates of aortic calcification between the two groups.

The covariates in the univariate analysis that reached statistical significance were selected for the further binary logistic regression analysis model except for DBP to examine the relationship between these risk factors and the development of postoperative SH. In the binary logistic regression model, preoperative calcium, iPTH, and ALP levels were independent predictors of postoperative SH. Patients with higher levels of iPTH and ALP and lower levels of serum calcium were found to be at a greater risk of developing SH after tPTX (Table 3). The adjusted odds ratio was 0.042, 10.452, and 46.245 for preoperative calcium, iPTH, and ALP, respectively. A higher weight of resected glands was associated with a greater risk of developing SH postoperatively, but not significantly.

**Figure 2:**
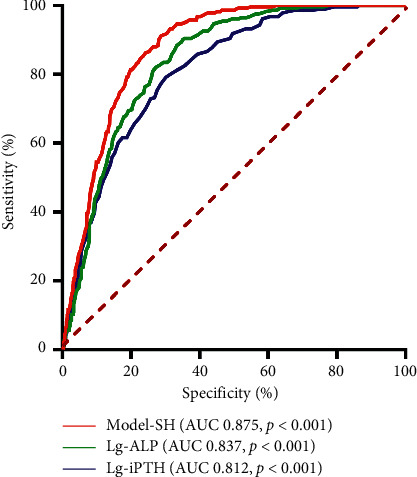
ROC curves for postoperative severe hypocalcemia by the binary logistic regression equation. This graph shows the ROC curves for preoperative Lg-iPTH (blue), Lg-ALP (green), and Model-SH (red). The prognostic factors in Model-SH include preoperative serum calcium, iPTH, and ALP. AUC for Lg-iPTH and Lg-ALP were 0.812 and 0.837, respectively. The cut-off values for Lg-iPTH and Lg-ALP were 3.21 and 2.32, respectively. AUC for Model-SH was 0.875 and the cut-off value was 3.25. The sensitivity and specificity of Lg-iPTH were 78.9% and 70.7%, respectively. The sensitivity and specificity of Lg-ALP were 90.5% and 64.7%, respectively. The sensitivity and specificity of Model-SH were 90.8% and 71.9%, respectively. Predictor = 0.738 *∗* Lg-ALP + 1.205 *∗* Lg-iPTH − (albumin − adjusted calcium) (all of the indexes were preoperative).

According to the binary logistic regression equation, receiver operating characteristic curves (ROC) were generated for the preoperative Lg-iPTH and Lg-ALP. The area under the curve was 0.812 for Lg-iPTH and 0.837 for Lg-ALP. The cut-off value was 3.21 for Lg-iPTH and 2.32 for Lg-ALP. Accordingly, the cut-off value of iPTH was 1,621.8 pg/ml and the cut-off value of ALP was 208.9 U/L. To evaluate the risk factors of SH postoperatively more accurately, a model combined of preoperative serum calcium, iPTH, and ALP levels was used. The area under the curve for these prognostic risk factors was 0.875. The sensitivity and specificity were 90.8% and 71.9%, respectively. As shown in Figure 2, the algorithm was as follows:

(1) $$\begin{array}{c} \text{predictor}=0.738*\text{Lg}-\text{ALP}+1.205*\text{Lg}-\text{iPTH}-\left(\text{albumin}-\text{adjusted calcium}\right). \end{array}$$

All of the indexes were preoperative.

The cut-off value was 3.25.

## 4. Discussion

The mean iPTH level (430 pg/mL) in China is higher than that in other DOPPS (dialysis outcomes and practice patterns study) regions (range 149–404 pg/mL), and the median Chinese facility reported that 19% of patients had serum iPTH levels of >600 pg/mL (range 0–15% in other DOPPS regions) [15, 16]. Data from dialysis and transplantation registration systems revealed that approximately 200 million patients were on dialysis in China, and the rate of SHPT patients is still rising. In our center, the prevalence of SHPT is 60.69%, and 22.41% of these patients underwent tPTX. The clinical effects of renal hyperparathyroidism include refractory pruritus, bone pain, muscular weakness, progressive soft tissue calcification, kidney stones, constipation, peptic ulcer disease, spontaneous long bone fracture, and even psychosis or dementia [4]. Parathyroidectomy is regarded as an ultimate therapeutic resource in patients with severe SHPT and can improve the bone metabolism and cardiovascular outcome of these patients [17–19].

This study established that the prevalence of postoperative SH among the study subjects was 25.9%, and the results are comparable to those of previous studies [20–22]. The pathogenesis of postoperative SH is multifactorial but the primary mechanism may involve the abrupt removal of iPTH stimulation after parathyroidectomy; hence, osteoclastic activity is reduced but continues to cause excess calcium and phosphate to pass into the bones [23]. Administration of PTH (1–34) after parathyroidectomy was able to prevent postsurgical hypocalcemia in primary hyperparathyroidism [24].

Previous studies have identified several predictors of the occurrence of early postoperative SH after parathyroidectomy, including the age of patients; preoperative calcium, ALP, vitamin D, and albumin levels; and weight of the resected glands [4, 20, 25, 26]. The present study revealed that patients in the SH group presented with a higher median preoperative ALP level than did the non-SH group (528.2 vs. 159.5 U/L, resp.). Using binary logistic regression analysis, high preoperative ALP had a positive predictive value for the development of early postoperative SH with an adjusted odds ratio (OR) of 10.452. An increased serum ALP level is a well-known feature in conditions of increased bone turnover such as primary and secondary hyperparathyroidism. In studies of patients with renal bone disease in which bone mineralization was measured along with serum ALP, improvement in bone mineralization along with a reduction in serum ALP was induced by active vitamin D compound treatment [27]. An elevated serum ALP level indicates an increase in bone metabolism and is associated with osteoporosis [28]. For patients with SHPT, the removal of the parathyroid glands corrects the abnormal bone metabolic status and, thus, calcium and phosphorus deposits in the bone; hence, the serum calcium levels drop dramatically. Therefore, the preoperative serum ALP level can be a predictor of postoperative SH. Patients with high preoperative ALP levels should undergo close monitoring of the serum calcium level in the early postoperative period, particularly those in the high dependency unit, to avoid the complication of early postoperative SH [29]. Postoperative empirical intravenous calcium supplementation should be administered in the immediate postoperative period and at a higher dose in high-risk patients. In our department, 5 g calcium gluconate was routinely prescribed for all patients immediately after the surgery. When oral intake is feasible, oral vitamin D and calcium supplementation should be started [30].

Preoperative iPTH levels are related to the severity of renal osteodystrophy. Therefore, a positive association between an increased preoperative serum iPTH level and postoperative SH is expected. In our study, patients in the SH group had a higher preoperative iPTH level than did the non-SH group (2,304.9 vs. 1,239.7 pg/mL). The results were consistent with previous studies [31–33].

None of the other factors evaluated, including sex, duration of hemodialysis, duration of renal failure, underlying kidney diseases, serum phosphorus levels, serum albumin levels, and vascular calcification, were predictive of the development of postoperative SH in renal patients with SHPT after tPTX. Univariate analysis revealed that the weight of the resected glands was associated with the risk of SH after tPTX, but binary logistic regression analysis revealed no significant difference. The overlapping effect of the weight of the resected glands and serum iPTH may explain the difference.

In the present study, we enrolled over 1,000 patients, and thus the sample size was larger than that in previous studies. Herein, we provided a diagnosis model for the risk of developing SH after tPTX in order to determine the need for preoperative interventions, such as calcium bicarbonate. According to the algorithm, the cut-off value was 3.25, meaning if the value of the predictor was more than 3.25, postoperative SH was likely diagnosed. Therefore, such patients could be given some prophylactic supplementation of calcium before the surgery.

However, some limitations of this study should be noted. First, the study was retrospective in nature, and the indication bias for surgery could not be avoided. In addition, as a single-center study in a tertiary center, the results might not be generalizable to all other dialysis patients, as patients in our center are mostly at the end-stage of the disease with severe symptoms and delayed surgical opportunity. Moreover, the supplementary volumes of calcium were not analyzed in this study, and additional factors should be included in future studies to reduce the potential effect of confounding variables.

In conclusion, most of our patients with SHPT were symptomatic preoperatively, and the most common clinical presentations were bone pain and pruritus. The significant predictors of early postoperative SH after tPTX in renal failure patients with SHPT included the preoperative iPTH and ALP levels. Such patients should be closely monitored in the early postoperative period in order to initiate prompt interventions to avoid SH.

## Figures and Tables

**Table 1:** The clinical characteristics of the patients who underwent tPTX.

*N*	1095
Male (*n*, %)	647 (59.1)
Age^b^	47.26 ± 10.86
Underlying kidney disease (*n*, %)	
CGN	645 (58.9)
DN	22 (2.0)
HTN	67 (6.1)
PKD	57 (5.2)
Others	76 (6.9)
Unknown	228 (20.8)
Comorbidity (*n*, %)	
Diabetes mellitus	45 (4.1)
Hypertension	872 (79.6)
Duration of dialysis (months)^a^	84 (60∼120)
SBP (mmHg)^b^	142.26 ± 20.88
DBP (mmHg)^b^	87.98 ± 15.04
Heartbeat (beats/min)^b^	82.88 ± 11.72
Clinical symptoms	
Bone pain (*n*, %)	873 (79.7)
Pruritus (*n*, %)	591 (54.0)
Laboratory test	
Preoperative iPTH (pg/ml)^a^	1448.4 (996.0∼2173.8)
Preoperative ALP (U/L)^a^	205.6 (117.0∼464.9)
Preoperative calcium (mmol/L)^b^	2.43 ± 0.24
Preoperative phosphorus (mmol/L)^b^	2.25 ± 0.56
Hemoglobin (g/L)^b^	107.67 ± 19.58
Albumin (g/L)^b^	41.89 ± 5.04

tPTX, total parathyroidectomy; CGN, chronic glomerulonephritis; DN, diabetic nephropathy; HTN, hypertensive nephropathy; PKD, polycystic kidney disease; SBP, systolic blood pressure; DBP, diastolic blood pressure. ^a^Median, Q1∼Q3. ^b^Mean ± SD.

**Table 2:** Preoperative indicators of patients in the SH and non-SH groups.

	Severe hypocalcemia		*p*=
	No (*n* = 811)	Yes (*n* = 284)	
Male (*n*, %)	475 (58.6)	172 (60.6)	0.575
Age (years)^b^	48.2 ± 10.8	44.6 ± 10.7	<0.001
Duration of dialysis (months)^a^	84 (60∼120)	84 (60∼120)	0.997
Diabetes mellitus (*n*, %)	39 (4.8)	6 (2.1)	0.055
Hypertension (*n*, %)	653 (80.3)	219 (78.4)	0.230
SBP (mmHg)^b^	142.0 (20.9)	142.9 (20.7)	0.528
DBP (mmHg)^b^	87.3 (14.7)	90.1 (15.8)	0.007
Heartbeat (beats/min)^b^	82.2 (11.6)	84.8 (11.9)	0.001
Clinical symptoms			
Bone pain (*n*, %)	625 (77.1)	248 (87.3)	<0.001
Pruritus (*n*, %)	461 (57.0)	130 (45.8)	0.001
Preoperative laboratory test			
iPTH (pg/ml)^a^	1239.7 (871.7∼1773.0)	2304.9 (1681.2∼2919.2)	<0.001
ALP (U/L)^a^	159.5 (102.8∼279.2)	528.2 (290.6∼848.3)	<0.001
Hemoglobin (g/L)^b^	109.3 (19.5)	103.2 (19.1)	<0.001
Albumin (g/L)^b^	41.9 (4.4)	41.7 (5.1)	0.563
Phosphorus (mmol/L)^a^	2.2 (1.9∼2.6)	2.2 (1.9∼2.5)	0.161
Calcium (mmol/L)^b^	2.5 (0.2)	2.4 (0.2)	<0.001
Surgical procedure			
Weights of resected glands (g)^a^	2.4 (1.5∼3.8)	3.2 (2.0∼5.0)	<0.001
Four glands resected (*n*, %)	668 (85.1)	247 (89.5)	0.285
Aortic calcification (*n*, %)	454 (57.4)	171 (61.3)	0.507

^a^Median, Q1∼Q3.^b^ Mean ± SD. Normal ranges: iPTH (12–88 pg/ml); ALP (35–100 U/L); hemoglobin (120–160 g/L for male and 110–150 for female); albumin (40–55 g/L); phosphorus (0.85–1.51 mmol/L); and calcium (2.15–2.55 mmol/L).

**Table 3:** Logistic regression analysis of the association between risk factors and postoperative severe hypocalcemia.

Variable	Estimate	SE	*p*	Adjusted OR	95% CI
Preoperative calcium	−3.181	0.412	<0.001	0.042	0.019∼0.093
Preoperative Lg-ALP	2.347	0.323	<0.001	10.452	5.555∼19.667
Preoperative Lg-iPTH	3.834	0.618	<0.001	46.245	13.783∼155.157

iPTH, intact parathyroid hormone; ALP, alkaline phosphatase; OR, odds ratio. Forward selection was used with *p* < 0.05 required to enter the equation.

## Data Availability

The data will be available under reasonable request and the responsive e-mail address is wenping@njmu.edu.cn.
